# Proteotypic classification of spontaneous and transgenic mammary neoplasms

**DOI:** 10.1186/bcr930

**Published:** 2004-10-06

**Authors:** Igor Mikaelian, Natalie Blades, Gary A Churchill, Karen Fancher, Barbara B Knowles, Janan T Eppig, John P Sundberg

**Affiliations:** 1 The Jackson Laboratory, Bar Harbor, Maine, USA

**Keywords:** cancer, epithelial to mesenchymal transition, keratin, mammary gland, neoplasia

## Abstract

**Introduction:**

Mammary tumors in mice are categorized by using morphologic and architectural criteria. Immunolabeling for terminal differentiation markers was compared among a variety of mouse mammary neoplasms because expression of terminal differentiation markers, and especially of keratins, provides important information on the origin of neoplastic cells and their degree of differentiation.

**Methods:**

Expression patterns for terminal differentiation markers were used to characterize tumor types and to study tumor progression in transgenic mouse models of mammary neoplasia (mice overexpressing *Neu *(*Erbb2*), *Hras*, *Myc*, *Notch4*, *SV40-TAg*, *Tgfa*, and *Wnt1*), in spontaneous mammary carcinomas, and in mammary neoplasms associated with infection by the mouse mammary tumor virus (MMTV).

**Results:**

On the basis of the expression of terminal differentiation markers, three types of neoplasm were identified: first, simple carcinomas composed exclusively of cells with a luminal phenotype are characteristic of neoplasms arising in mice transgenic for *Neu*, *Hras*, *Myc*, *Notch4*, and *SV40-TAg*; second, 'complex carcinomas' displaying luminal and myoepithelial differentiation are characteristic of type P tumors arising in mice transgenic for *Wnt1*, neoplasms arising in mice infected by the MMTV, and spontaneous adenosquamous carcinomas; and third, 'carcinomas with epithelial to mesenchymal transition (EMT)' are a characteristic feature of tumor progression in *Hras-*, *Myc-*, and *SV40-TAg-*induced mammary neoplasms and PL/J and SJL/J mouse strains, and display *de novo *expression of myoepithelial and mesenchymal cell markers. In sharp contrast, EMT was not detected in papillary adenocarcinomas arising in BALB/cJ mice, spontaneous adenoacanthomas, neoplasms associated with MMTV-infection, or in neoplasms arising in mice transgenic for *Neu *and *Wnt1*.

**Conclusions:**

Immunohistochemical profiles of complex neoplasms are consistent with a stem cell origin, whereas simple carcinomas might originate from a cell committed to the luminal lineage. In addition, these results suggest that the initiating oncogenic events determine the morphologic features associated with cancer progression because EMT is observed only in certain types of neoplasm.

## Introduction

Architectural and cytological patterns have been the basis for mammary tumor categorization for more than a century [1]. Over the past 20 years, immunohistochemistry has added a molecular dimension to the categorization of mammary neoplasms: altered expression of oncogenes, oncosuppressor genes, hormone receptors, and cytoskeletal proteins have been identified as useful indicators of disease outcome. Gene microarray analysis has confirmed the importance of many of these histological prognosticators [2-4], which – in conjunction with clinical features such as lymph node status, tumor size, and tumor grade [5] – are undeniably useful. However, these prognosticators do not seem to be useful to identify the pathways that lead to tumorigenesis and cancer progression.

Transgenic mouse models of mammary cancer have added considerably to our knowledge of human breast tumorigenesis. More specifically, in addition to dissecting the molecular mechanisms underlying mammary development and the initial steps of mammary neoplastic transformation, mouse models have shown that activation of a particular pathway is reflected in specific histologic patterns [6,7].

In view of the evidence that terminal differentiation markers are important pathway markers [7] and prognostic predictors [4,8-10], we compared the expression of terminal differentiation markers and some morphologic features of spontaneous and transgene-induced mammary tumors in mice. This study corroborates molecular [2,3,11-14] and immunohistochemical [4,7,15] studies in human cancer patients and mouse models of human cancer recommending that, in addition to the broadly accepted and well-documented use of architectural features [6,7,16-19], diagnostic criteria should also take into account the cell types into which neoplastic cells differentiate.

We have identified neoplastic cells with luminal, myoepithelial, and mesenchymal phenotypes. Myoepithelial differentiation is 'constitutive' to specific tumor types, for example adenosquamous carcinomas, adenomyoepitheliomas, and adenocarcinomas associated with the active mouse mammary tumor virus (MMTV) and the wingless-related MMTV integration site 1 (*Wnt1*) transgene. In contrast, in neoplasms dependent on the myelocytomatosis oncogene (*Myc*), Harvey rat sarcoma viral oncogene 1 (*Hras*), and SV40 T antigen (*SV40-TAg*) transgenes, myoepithelial differentiation is an important event in the specific type of tumor progression characterized by epithelial to mesenchymal transition (EMT). The occurrence in some transgenic models of distinct proteotypic expression patterns, defined as groups of neoplasms expressing a specific set of terminal differentiation markers, suggests an as yet unreported degree of variability in transgenic models, perhaps reflective of the diversity of second-hit mutations in these tumors. Finally, specific types of spontaneous mammary tumors are histologically and immunohistochemically indistinguishable from some of the transgene-induced mammary tumor models. This provides the background information to test the hypothesis that tumor phenotype is a predictor of tumor genotype.

## Methods

### Mice

The pathology database [20] of The Jackson Laboratory was searched from 1987 to 2001 for spontaneous mammary tumors in production and research colonies. Slides were reviewed and categorized on the basis of standardized criteria [17-19,21-23].

In addition, newborns of the following strains were obtained from The Jackson Laboratory Induced Mutant Resource (Bar Harbor, ME): C57BL/6J-Tg(WapTAg)1Knw, FVB/N-Tg(WapNotch4)10Rnc/J, B6SJL-Tg(Wnt1)1Hev/J (hereafter abbreviated B6SJL-Wnt1), FVB/NJ-Tg(Wnt1)1Hev/J (hereafter abbreviated FVB-Wnt1), FVB/N-Tg(MMTVneu)202Mul/J, FVB/N-Tg(WapMyc)212Bri/J, FVB/N-Tg(WapHRAS)69Lln Y^SJL^/J, and FVB/NJ-Tg(MMTVTGFA)254Rjc/J and B6D2-Tg(MMTVTGFA)254Rjc/J. Mice were weaned at 3 weeks of age and genotyped by polymerase chain reaction. Detailed information on the genotyping protocols and breeding conditions are available online at . These mice were aged until they developed mammary masses, which were fixed by immersion in Fekete's acid–alcohol–formalin. Slides were sectioned at 5–6 μm and stained with hematoxylin and eosin (H&E).

### Immunohistochemistry

Consecutive sections (5–6 μm thick) on Superfrost Plus slides (Fisher Scientific, Fair Lawn, NJ) of paraffin-embedded neoplasms were immunolabeled for α-smooth muscle actin (SMA; Sigma, St Louis, MO), keratin 1 (KRT2-1, hereafter abbreviated K1; BabCo, Richmond, CA), keratin 5 (KRT2-5, hereafter abbreviated K5; BabCo), keratin 6 (KRT2-6, hereafter abbreviated K6; BabCo), keratin 10 (K1-10, hereafter abbreviated K10; Babco), keratin 13 (KRT13, hereafter abbreviated K13; Sigma), keratin 14 (KRT1-14, hereafter abbreviated K14; BabCo), keratin 17 (KRT1-17, hereafter abbreviated K17; PA Coulombe, The Johns Hopkins University, Baltimore, MD) [24], keratins 8/18 (KRT8 and KRT18, hereafter abbreviated K8/18; Progen, Heidelberg, Germany), vimentin (Biomeda, Foster City, CA), filaggrin (BabCo), involucrin (Babco), loricrin (Babco), and trichohyalin (T-T Sun, The New York University School of Medicine, New York, NY) as described previously [25]. All these antibodies were mouse-specific except the antibodies against K13, trichohyalin, and vimentin, which were raised against human proteins, the antibody against K8/18, which was raised against bovine keratins, and the antibody against SMA, which was raised against avian proteins. The anti-SMA antibody recognizes aortic α2 smooth muscle actin (ACTA2) and enteric γ2 smooth muscle actin [26].

In brief, the method was as follows. Deparaffinized slides were gradually hydrated. Endogenous peroxidase activity was quenched by incubation with 3% hydrogen peroxide in methanol for 20 min at room temperature (20–25°C). Heat-mediated antigen retrieval in citrate buffer at pH 6.0 was used for the antibodies targeted at K8/18 and vimentin. Slides were washed and were incubated for 30 min with blocking serum (10% normal fetal calf serum diluted in phosphate-buffered saline). Excess blocking serum was blotted and the slides were incubated overnight at 4°C with primary antibodies diluted in phosphate-buffered saline. Secondary biotinylated anti-mouse or anti-rabbit antibody was applied for 30 min at room temperature, followed by incubation with the avidin-biotin complex (45 min). The reaction was developed with the substrate diaminobenzidine (Sigma) and the slides were counterstained with Mayer's hematoxylin. All spontaneous tumors were labeled for MMTV gp27 Gag, gp36 Env, and gp52 Env [27]. A mouse was identified as positive for MMTV if immunolabeling for at least one of the markers was detected in the tumor or nearby tissues.

Images were captured with a DP70 digital camera (Olympus, Melville, NY) on an Olympus BX41 microscope and color enhanced and balanced for contrast with Photoshop 6.0 (Adobe, San Jose, CA). Additional photomicrographs of H&E-stained and immunolabeled slides are archived in the Mouse Tumor Biology Database, where they can be viewed on-line at [28].

### Slide interpretation and scoring of immunolabeling intensity

Cell types were defined on the basis of a previous study that evaluated the expression of the same terminal differentiation markers in the developing mammary gland [29].

Expression of K5, K14, K17, and/or SMA was associated with a direct contact with a basement membrane and the morphology of a myoepithelial cell defined myoepithelial cell differentiation. Absence of labeling for any of the markers, or labeling for K8/18 along with a polygonal morphology, defined luminal cells.

Image analysis (Photoshop 6.0; Adobe, San Jose, CA) was used to evaluate the ratio of spindloid neoplastic cells on H&E-stained sections on all tumors with EMT. EMT was considered significant when more than 1% of the neoplasm was composed of neoplastic cells having a spindloid to fusiform shape and blending into the stroma. These cells had an oval to elongated nucleus with less clumped chromatin than epithelial neoplastic cells. Their cytoplasm was more acidophilic than epithelial neoplastic cells.

Squamous differentiation was defined by the presence of one or more of the following features: the formation of a large core of cornified material, the formation of keratin pearls, the cornification of individual cells, or the presence of trichohyalin granules or keratohyalin granules. Squamous differentiation was confirmed by immunohistochemical expression of at least one of the following markers in the areas of squamous differentiation: K1, K6, K10, filaggrin, trichohyalin, involucrin, or loricrin, none of which is normally expressed in cycling mammary glands [29].

The intensity of immunolabeling was scored on a scale of 0 to 3 (immunolabeling levels: 0 = none; 1 = weak; 2 = moderate; 3 = intense). The grade for each cell type was the product of average labeling intensity and the relative percentage of cells (0%, 0; up to 1%, 1; 2–5%, 2; 6–25%, 3; 26–50%, 4; more than 50%, 5) labeled at the predominant intensity. The outcome for each neoplasm was the sum of the grades for each cell type. If immunolabeling was detected in less than 10 cells in a tumor, the tumor was considered to be negative for this marker.

### Morphologic criteria

All tumors were evaluated (presence/absence) on H&E-stained sections for myoepithelial differentiation, EMT, squamous differentiation, vascular invasion, proteinaceous or lipid droplets in neoplastic cells, and ductal differentiation (defined as tubular structures lined by a basal layer of cells with myoepithelial differentiation and a luminal layer of cuboidal cells). Type P tumors are neoplasms composed of a branching network of blind ducts lined by an epithelium at least two cells thick and terminated by structures resembling the terminal end buds of the pubertal mammary gland [7]. Macrocysts are large cysts lined by an epithelium one or two cells thick that occasionally forms small glands. Lactation-responsive plaques are discoid masses composed of contoured and anastomosed mammary acini at the periphery and ducts at the center.

## Results

### Histologic tumor types (Table 1)

Tumors were categorized histologically using the recommendations of the Mouse Models of Human Cancers Consortium categorization scheme [17-19] and the specific nomenclature applying to certain models [22] or tumor types [21,23] when applicable.

**Figure 1:**
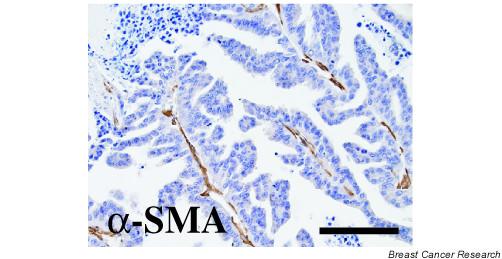
Papillary adenocarcinoma lined by an epithelium one cell thick in a BALB/cJ mouse: neoplastic cells lack myoepithelial differentiation. Myofibroblasts are present in the stroma of the neoplasm. Scale bar, 80 μm.

**Figure 2:**
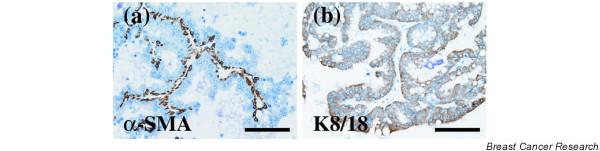
Papillary adenocarcinoma lined by an epithelium two cells thick: there is myoepithelial differentiation of the basal layer **(a)**, whereas suprabasal cells have a luminal phenotype **(b)**. Scale bar, 80 μm.

**Figure 3:**
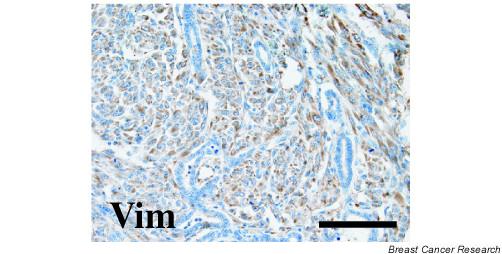
Epithelial to mesenchymal transition (EMT) in a spontaneous neoplasm: neoplastic cells with a spindloid phenotype are labeled strongly for vimentin in a glandular carcinoma with EMT in an SJL/J mouse. Scale bar, 80 μm.

**Figure 4:**
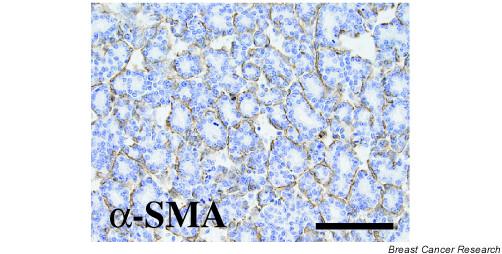
Microacinar carcinoma in a C3H/HeJ mouse infected by the mouse mammary tumor virus. Microacinar carcinomas are characterized by a microacinar pattern with prominent myoepithelial differentiation. Scale bar, 80 μm.

**Figure 5:**
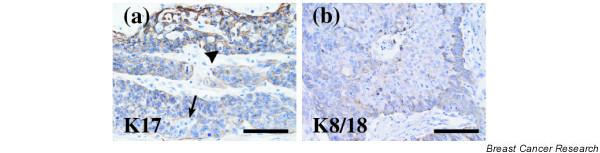
Adenomyoepithelioma in a BALB/cJ mouse: individual cells **(a**, arrowhead**)**, clusters of cells (a, arrow), and occasionally large areas **(b) **are not labeled by antibodies against terminal differentiation markers of myoepithelial (a) and luminal cells (b) of the mammary gland. Scale bar, 80 μm.

**Figure 6:**
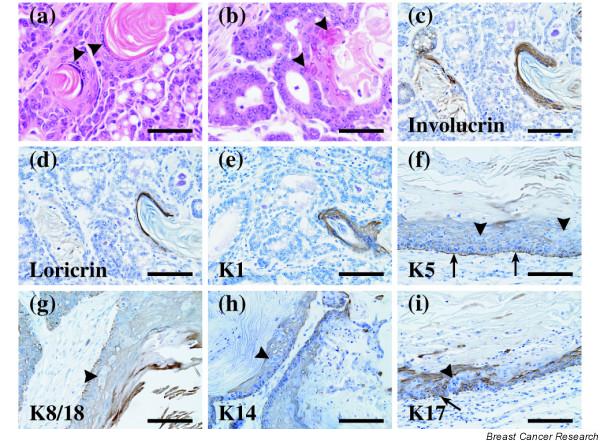
Adenosquamous carcinoma in a BALB/cJ mouse: squamous differentiation is commonly associated with the presence of keratohyalin **(a, **arrowhead**)** or trichohyalin **(b**, arrowhead**)** granules. Squamous differentiation is 'complete', with expression of markers of all layers of the epidermis **(c-e)**. Individual cells and small clusters of cells fail to express, or express only weakly, any terminal differentiation marker **(f-i**, arrowheads**)**, whereas myoepithelial differentiation is observed in the areas of squamous differentiation (f, i, arrows). Scale bars, 50 μm (a, b) and 80 μm (c-i).

Spontaneous tumors were papillary adenocarcinomas with papillae lined by a single layer of cells (*n *= 6; Fig. 1), papillary adenocarcinomas with papillae lined by two layers of cells (*n *= 2; Fig. 2), glandular adenocarcinomas with EMT (*n *= 4; Fig. 3), microacinar adenocarcinomas in C3H/HeJ mice (*n *= 8; Fig. 4), type P tumors in C3H/HeJ mice (*n *= 3), adenomyoepitheliomas in BALBc/J mice (*n *= 5; Fig. 5), and adenosquamous carcinomas (*n *= 7; Fig. 6).

**Figure 7:**
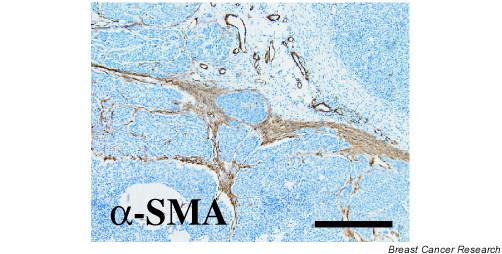
Solid carcinoma from a mouse transgenic for *Hras*: expression of α-smooth muscle actin in carcinomas is restricted to stromal myofibroblasts and vascular smooth muscle fibers. A similar observation was made for carcinomas arising in mice transgenic for *Neu *(*Erbb2*) and *Notch 4 *and in most mice transgenic for SV40-TAg (not shown). Scale bar, 320 μm.

**Figure 8:**
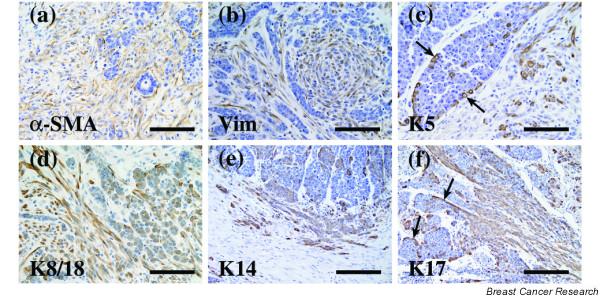
Epithelial to mesenchymal transition (EMT) in a mouse transgenic for *Myc*: EMT is associated with expression of terminal differentiation markers of mesenchymal **(a,b)**, myoepithelial **(c,e,f) **and luminal **(d) **cells, with *de novo *acquisition of a myoepithelial phenotype (c,e,f, arrows) by neoplastic cells. Scale bars, 80 μm (a-d) and 160 μm (e, f). '*De novo*' acquisition of the myoepithelial phenotype is defined as neoplastic cells displaying a myoepithelial phenotype in a portion of the neoplasm limited to the vicinity of the areas of EMT, whereas myoepithelial differentiation was lacking in the rest of the neoplasm (not shown).

**Figure 9:**
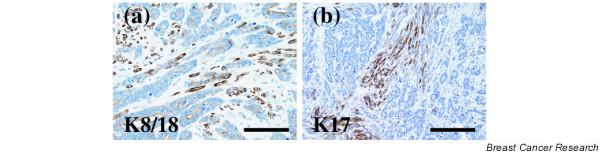
Epithelial to mesenchymal transition (EMT) in a mouse transgenic for SV40-TAg: EMT is associated with increased expression of luminal **(a) **and myoepithelial **(b) **terminal differentiation markers. Scale bars, 80 μm (a) and 160 μm (b).

**Figure 10:**
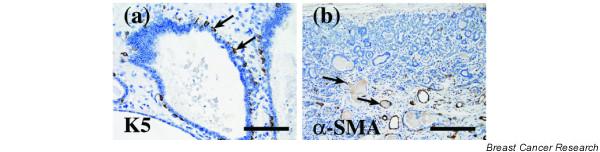
Neoplasms from *Tgfa*-transgenic mice: myoepithelial differentiation is present in macrocysts **(a**, arrows<) and in lactation-responsive plaques **(b**, arrows**)**. Scale bars, 80 μm (a) and 160 μm (b).

**Figure 11:**
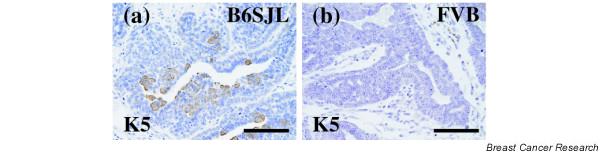
Influence of the background strain on protein expression in type P tumors: the expression of K5 **(a,b) **and K8/18 (not shown) is higher, and squamous differentiation (not shown) is more prominent in B6SJL-*Wnt1 *than in FVB/NJ-*Wnt1 *mice. Scale bar, 80 μm.

All *Hras*-induced (*n *= 4; Fig. 7) and *Neu*-induced (*n *= 10) tumors as well as some *Myc*-induced (*n *= 4; Fig. 8c,8e,8f) and SV40-TAg-induced tumors (data not shown) had a solid pattern. Some SV40-TAg-induced (*n *= 5; Fig. 9a,9b) and *Myc*-induced tumors (*n *= 9; Fig. 8a,8b,8d) as well as all *Notch4*-induced tumors (*n *= 6) had a glandular pattern. Some *Myc*-induced (*n *= 13; Fig. 8), SV40-TAg-induced (*n *= 3; Fig. 9), and *Hras*-induced (*n *= 3) carcinomas displayed areas of EMT. Some SV40-TAg-induced tumors (*n *= 4) had a papillary pattern with papillae lined by epithelial cells that piled up in a disorderly fashion (data not shown). Preneoplastic lesions of *Tgfa*-transgenic mice consisted of macrocysts (*n *= 8; Fig. 10a) and lactation-responsive plaques (*n *= 3; Fig. 10b). All *Wnt1*-induced tumors (*n *= 13; Fig. 11) exhibited a type P tumor pattern.

All spontaneous type P tumors (*n *= 9), all microacinar carcinomas in C3H/HeJ mice (*n *= 8), and one of two papillary carcinomas in one C3H/HeJ mouse showed immunolabeling for MMTV (data not shown). Proteinaceous and/or lipid secretion was identified in a small number of tumors in most transgenic models and spontaneous tumor types (Fig. 2a,2b), although it was observed most consistently in macrocysts and in adenosquamous carcinomas (Fig. 6a,6c,6d,6e).

### Terminal differentiation proteins expression patterns

The neoplasms were categorized into three groups: those with a pure luminal phenotype (simple carcinomas), those that constitutively expressed myoepithelial and luminal markers, and those that were characterized by EMT. Hampe and Misdorp [30] defined the term complex as 'any type of neoplasm or proliferation composed of cells resembling both secretory epithelial and myoepithelial cells'. We therefore used the term 'constitutively complex carcinomas' to indicate the tumors in which luminal and myoepithelial cells were arranged as they would be in a normal mammary gland, suggesting that these two types of cells originated from the same progenitor cell. The tumors that expressed myoepithelial markers in the areas of EMT only are designated 'acquired complex carcinomas' to indicate that myoepithelial differentiation was absent in the areas representing the original phenotype of the neoplasms, before the occurrence of EMT.

**Figure 12:**
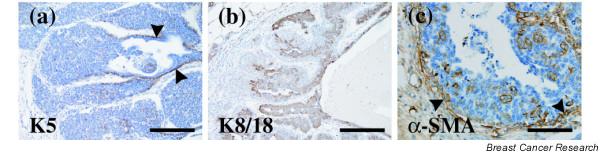
Type P tumors arise in mice infected by the mouse mammary tumor virus. Myoepithelial differentiation is prominent in ductal areas **(a**, arrowheads**)**, whereas suprabasal cells have a luminal phenotype and label for K8/18 **(b)**. Clusters and individual suprabasal cells are labeled by antibodies against α-smooth muscle actin **(c) **and in cells with myoepithelial differentiation (arrowheads). Scale bars, 320 μm (a), 160 μm (b) and 80 μm (c).

Tumor categorization was then redesigned to take into account not only the architecture of the neoplastic process [17-19] but also myoepithelial differentiation and EMT, which were not always apparent on H&E-stained sections. Keratins 5, 14, and 17, which are generally considered to be markers of myoepithelial cells [4], were occasionally identified in suprabasal cells in a variety of neoplasms (Figs 6f,6h,6i,8c,8e,8f,11a). α-Smooth muscle actin, another marker of myoepithelial cells, was expressed by suprabasal cells only in MMTV-associated and *Wnt1*-induced type P tumors (Fig. 12c), a feature previously noted by Li and colleagues [13].

Neoplasms with a pure luminal phenotype (simple carcinomas) were identified in all transgenic models of mammary carcinogenesis (Fig. 7, Table 1) with the notable exception of *Wnt1*-induced carcinomas that had a constitutively complex phenotype. Most (six of nine) glandular carcinomas arising in mice transgenic for Myc showed a few areas of myoepithelial differentiation that were interpreted as early EMT rather than the neoplasms arising from a progenitor cell common to the luminal and myoepithelial phenotypes; hence these neoplasms with minimal myoepithelial differentiation were categorized as 'simple' carcinomas. The only spontaneous neoplasms composed exclusively of cells with a luminal phenotype were four of six papillary carcinomas that, on H&E sections, were characterized by papillae lined by an epithelium one cell thick.

Constitutively complex carcinomas consisted of spontaneous papillary carcinomas lined by an epithelium two cells thick (Fig. 2), microacinar adenocarcinomas arising in MMTV-infected C3H/HeJ mice (Fig. 4), type P tumors of MMTV-infected C3H/HeJ mice (Fig. 12) and *Wnt1*-transgenic mice (Fig. 11), adenomyoepitheliomas (Fig. 5), adenosquamous carcinomas (Fig. 6), and lactation-responsive plaques and most (six of eight) macrocysts of *Tgfa*-transgenic mice (Fig. 10). Neoplastic cells with a myoepithelial phenotype were abundant and formed an almost continuous layer surrounding cells with a luminal phenotype in microacinar carcinomas (Fig. 4) and in spontaneous papillary carcinomas lined by an epithelium two cells thick (Fig. 2a). In type P tumors, myoepithelial differentiation was most prominent in the areas of ductal metaplasia and was minimal or absent in the frond-like areas of the neoplasms (Fig. 12a). In adenosquamous carcinomas, myoepithelial differentiation was most prominent in the areas of squamous differentiation (Fig. 6f,6i), and was often absent in the glandular areas.

In adenomyoepitheliomas (*n *= 5), large proportions of suprabasal cells were labeled for K5, K6, K8/18, K14, and K17, with K1 being expressed in only two tumors, and with duct formation and cornification being identified in four and three neoplasms, respectively. The morphology of neoplastic cells in adenomyoepitheliomas varied, ranging from fusiform to cuboidal. The proteotypic pattern of these cells is not characteristic of any specific cell type, and these cells were categorized as 'cells with myoepithelial differentiation' when in contact with a basement membrane and as 'luminal cells' when not in contact with a basement membrane. Neoplastic cells in adenomyoepitheliomas were difficult to differentiate from stromal myofibroblasts and, as reported earlier [31], they were negative for SMA.

In lactation-responsive plaques of *Tgfa*-transgenic mice, myoepithelial differentiation was most prominent in the areas of ductal differentiation located at the center of these lesions (Fig. 10b). The peripheral portions of lactation-responsive plaques, which morphologically resemble mammary acini, generally lacked myoepithelial differentiation. Macrocysts were predominantly composed of cells with a luminal phenotype (Table 1) that occasionally rested directly on the basement membrane. Segmental portions of six of eight macrocysts had myoepithelial cells (Fig. 10a).

Immunohistochemistry indicated that the descriptive term 'papillary carcinoma' encompasses a variety of neoplasms. First, most (four of six) spontaneous papillary carcinomas of BALB/cJ mice (Fig. 1) and all (four of four) papillary carcinomas with a pseudostratified epithelium in SV40-TAg-transgenic mice were exclusively composed of cells with a luminal phenotype (simple carcinomas). Cells with myoepithelial differentiation, identified in two of six spontaneous papillary carcinomas of BALB/cJ mice, were scarce and might represent entrapped remnants of the normal mammary gland, because they were located at the periphery of the neoplasm. In spontaneous papillary adenocarcinomas characterized by two layers of cells lining neoplastic papillae, the basal layer was continuous and had a myoepithelial phenotype (Fig. 2a), whereas cells of the luminal layer expressed K8/18 (Fig. 2b).

Type P tumors, adenomyoepitheliomas, and adenosquamous carcinomas were characterized by the presence of subpopulations of cells with a high mitotic index that did not express terminal differentiation markers, or expressed them only weakly. In type P tumors, these cells were grouped at the ends of the fronds, the morphology and immunohistochemistry of which mimicked terminal end buds of the pubertal mammary gland [29].

Adenomyoepitheliomas (Fig. 5a,5b) and adenosquamous carcinomas (Fig. 6f,6g,6h,6i) were populated by individual cells and clusters of cells with a large open-faced nucleus and a moderate amount of pale cytoplasm with the appearance of ground glass. These cells were haphazardly distributed in the solid areas of adenomyoepitheliomas and in the viable epithelium enclosing cornified debris in adenosquamous carcinomas. Similar populations of neoplastic cells with low expression of terminal differentiation markers were not detected in the other neoplasms.

Carcinomas in B6SJL-*Wnt1*-and FBV-*Wnt1*-transgenic mice were essentially similar and had the classical appearance of a type P tumor. However, they differed slightly with regard to their proteotypic patterns: squamous differentiation and expression of K5 and K8/18 was higher in B6SJL-*Wnt1*-than in FBV-*Wnt1*-transgenic mice (Table 1; Fig. 11a,11b).

### Tumor progression

EMT, an important morphologic marker of tumor progression [32,33], was detected in 23 tumors. It was most commonly observed in *Myc*-induced carcinomas although it was also identified in *Hras*-and in SV40-TAg-induced carcinomas, and in carcinomas spontaneously arising in SJL/J and PL/J mice. Various proportions of neoplastic cells with a mesenchymal phenotype expressed vimentin (Figs 3, 8b), K5 (Fig. 8c), K14 (Fig. 8e), and K17 (Figs 8f, 9b). These cells consistently expressed SMA but could not be differentiated from myofibroblasts on the basis of the expression of this antigen alone (Fig. 8a). EMT was also associated with increased expression of K8/18 (Figs 8d, 9a). In most cases (20 of 23), a small number of cells with a myoepithelial phenotype were present in the glandular or solid areas in the immediate vicinity of the areas of EMT (Fig. 8c,8f).

Morphologic progression was also observed in microacinar adenocarcinomas associated with MMTV infection in C3H/HeJ mice, where it was characterized by focal to multifocal acquisition of a solid pattern. Acquisition of the solid pattern was associated with a loss of immunolabeling for MMTV proteins (data not shown) by neoplastic cells, but the expression of terminal differentiation markers remained unaltered.

*Neu*-induced carcinomas did not display a morphologic alteration suggestive of tumor progression, although the tumors evaluated included both metastatic and non-metastatic neoplasms (data not shown). The markers tested in this study did not segregate the three cell types classically described in *Neu*-and *Hras*-induced tumors [7]. EMT was not observed in *Notch4*-induced carcinomas, although the glandular pattern of these neoplasms is reminiscent of *Myc*-and SV40-TAg-induced carcinomas.

### Pathway pathology

Three histologic features of mammary tumors have recently been related to the activation of specific pathways: myoepithelial differentiation has been associated with activation of the Wnt pathway [7], squamous metaplasia has been attributed to β-catenin stabilization [34-37], and alveolar differentiation and milk secretion are dependent on signal transducer and activator of transcription 5a [38]. As expected, all *Wnt1*-induced carcinomas and spontaneous type P tumors displayed myoepithelial differentiation. In addition, myoepithelial differentiation, as assessed by routine H&E histology or immunohistochemistry, was identified in spontaneous papillary carcinomas with a bi-stratified epithelium, MMTV-associated microacinar carcinomas, adenomyoepitheliomas, and adenosquamous carcinomas.

Squamous metaplasia associated with expression of the full array of terminal differentiation markers of the suprabasal layers of the epidermis was found only in adenosquamous carcinomas (Fig. 6a,6b,6c,6d,6e,6f,6g,6h,6i). However, squamous metaplasia was also identified in some MMTV-associated (7 of 9) and *Wnt1*-induced (12 of 13) type P tumors, *Myc*-induced carcinomas (7 of 19), adenomyoepitheliomas (3 of 5), and spontaneous carcinomas exhibiting EMT (1 of 4). In spite of areas histologically consistent with squamous differentiation, MMTV-associated and transgene-induced type P tumors express only a limited set of suprabasal markers of the epidermis (Table 1). For example, K1 and K10, two markers of the stratum spinosum, were seldom expressed (4 of 30 and 6 of 30 carcinomas, respectively) in neoplasms other than adenosquamous carcinomas that exhibited cornification, whereas involucrin, a marker of the stratum corneum, was expressed in 19 of 30 such neoplasms.

## Discussion

In the present study, interpretation of immunohistochemistry results determined the following: first, that expression of terminal differentiation markers categorizes spontaneous and transgenic models of mammary neoplasia into 'simple' carcinomas, 'complex' carcinomas, and carcinomas with EMT; second, that most neoplasms are composed of multiple cell populations; and third, that some transgenic and spontaneous neoplasms are histologically and immunohistochemically indistinguishable, and they provide an opportunity to test the hypothesis that tumor phenotype predicts tumor genotype [6,7].

### Proteotypic patterns: luminal, luminal/myoepithelial, and luminal with EMT

Like human breast tumors [14], most mouse mammary tumors are exclusively composed of neoplastic cells with a luminal phenotype, suggesting that they developed from a progenitor cell committed to the luminal lineage. Progression to EMT through a myoepithelial stage was observed in *Myc*-, *Hras*-, and SV40-TAg-induced carcinomas, a subset of spontaneous mammary carcinomas in PL/J and SJL/J mice, and has also been reported in mice transgenic for *Tgfa *[39] and matrix metalloproteinase 3 [40]. Three major pathways have been implicated in EMT: the pathway controlled by the activation of Hras and Src, the transforming growth factor β pathway, and the Wnt pathway [32]. Evaluation of the expression of target genes of each of these pathways is needed to determine the molecular mechanisms associated with EMT in mouse models of mammary carcinogenesis.

Type P tumors and microacinar carcinomas constitutively express myoepithelial markers, whereas expression of these markers is an indicator of progression in *Myc*-, *Hras*-, and SV40-TAg-induced carcinomas, as it is in some types of human breast cancer [4,15,41]. As a consequence, misinterpretations are likely to occur if tumors are evaluated for EMT with whole-tumor methodologies such as gene or protein array technologies.

The genetic background of transgenic mouse models of mammary tumors might account for differences in the penetrance, latency, and phenotype of mammary proliferating lesions [42-44]. The histological phenotype of tumors arising in *Wnt1*-transgenic mice was similar, regardless of the background. However, the background strain influenced the proteotypic pattern of these tumors. This observation emphasizes the importance of the background strain in genetically engineered mice, suggests the presence of tumor modifier genes, and indicates possible similar differences in humans.

### Cell populations

The presence of cells negative or weakly positive for terminal differentiation markers establishes the presence of compartments of less differentiated cells in specific types of mammary carcinomas. This observation supports the hypothesis that undifferentiated cells, so-called 'mammary stem' cells, are the source of mammary tumors [45,46]. The histologic phenotype and the proteotypic pattern of type P tumors constitutes the most striking evidence for this hypothesis: the fronds in these carcinomas caricatured terminal end buds of the pubertal mammary gland, one of the compartments in which mammary stem cells are found [47,48]. Gene array data also support a stem cell origin for neoplasms arising in mice transgenic for *Wnt1 *[13]. In adenomyoepitheliomas and adenosquamous carcinomas, the location of cells that did not express terminal differentiation markers is suggestive of a ductal origin for these neoplasms. Interestingly, a second compartment of mammary stem cells is located in ductal suprabasal cells [49].

In addition to viable cells, a large proportion of neoplasms contained areas of cornification and necrosis. This heterogeneous group of non-viable cells has recently been named 'non-tumorigenic cancer cells' [50]. These cells need to be differentiated from neoplastic cells with self-renewal potential, including those cells with invasive or metastatic potential. Because the populations of 'non-tumorigenic cancer cells' might account for a significant proportion of some neoplasms, they might interfere with studies aiming at evaluating RNA or protein expression or genetic damage at the whole-tumor level.

### Pathway pathology

Cardiff and colleagues [6] initially proposed that tumor architecture and terminal differentiation markers can predict tumor genotype. The concept has subsequently been refined [7] and is supported by molecular data [12,13,51]. The present study confirmed that simple carcinomas with a solid pattern are typical of tumors driven by the *Neu *and *Ras *pathways, whereas type P tumors are a feature of neoplasms arising in mice transgenic for *Wnt1*. That type P tumors are also found in MMTV-infected mice was expected because *Wnt1 *is activated in 75% of mammary carcinomas arising in C3H/HeJ mice [52], and this observation supports the validity of the pathway pathology hypothesis.

Although promising, the pathology pathway concept has some limitations that are illustrated by our data. For example, *Myc*-induced carcinomas express large amounts of whey acidic protein and casein β [53], which should correlate with histologic evidence of proteinaceous secretion. However, only 5 of 20 *Myc*-induced carcinomas examined in this study had evidence of proteinaceous or lipid secretion. In addition, the pathology pathway concept predicts that *Myc*-, *Hras*-, and *Neu*-induced carcinomas should share similar pathways, distinct from those of *Wnt1*-induced carcinomas. However, *Myc*-and *Wnt1*-induced neoplasms are independent of cyclin D1, whereas *Hras*-and *Neu*-induced carcinomas are dependent on cyclin D1 [12,54], indicating that tumor phenotype and tumor genotype are not consistently matched.

## Conclusions

The observations that mammary neoplasms of mice transgenic for *H-Ras*, *Myc*, *Neu*, *Notch4 *and *SV40-Tag*, and that papillary carcinomas of BALB/cJ mice are exclusively composed of cells with a luminal phenotype, are consistent with the hypothesis that these neoplasms arise from a cell committed to the luminal lineage. The fact that adenomyoepitheliomas, adenoacanthomas, microacinar carcinomas, and type P tumors have a complex phenotype is consistent with a mammary stem cell origin. In addition, it is apparent that cancer progression in mammary carcinomas of mice transgenic for *H-Ras*, *Myc*, and *SV40-Tag *is often associated with EMT. However, EMT does not occur in mammary tumors of mice transgenic for *Tgfa*, *Neu*, *Notch4*, and *Wnt1*, which indicates that the morphologic features associated with cancer progression are determined by the initiating oncogenic events.

## Competing interests

The authors declare that they have no competing interests.

## Abbreviations

B6SJL-Wnt1 = B6SJL-Tg(Wnt1)1Hev/J; EMT = epithelial to mesenchymal transition; FVB-Wnt1 = FVB/NJ-Tg(Wnt1)1Hev/J; H&E = hematoxylin and eosin; Hras = Harvey rat sarcoma viral oncogene 1; K = keratin; MMTV = mouse mammary tumor virus; Myc = myelocytomatosis oncogene; SMA = α-smooth muscle actin; Wnt1 = wingless-related MMTV integration site 1.

## Figures and Tables

**Table 1:** Proteotypic Classification of Spontaneous and Transgenic Mammary Neoplasms Based on Terminal Differentiation Markers^a^.

Type	Strain/* Transgene*^b^	Main pattern^c^	Myoepithelial differentiation^d^	EMT (%)^e^	K1	K5	K6	K8/18	K10	K14	K17	FLG	IVL	LOR	THH	SMA	VIM
Simple	FVB-*Myc*	Glandular	6/9	4/9 (<1%)		+	+	+		++	+						
	FVB-*Notch4*	Glandular	0/6	0				+		+	+						
	C57BL6/J-Tag	Glandular	1/5	0				+		+	+						
	BALB/cJ (mostly)	Papillary, 1 cell thick	2/6	0		+	+	++		++	++						
	C57BL6/J-Tag	Papillary	0/4	0				+		+	+						
	FVB-*Myc*	Solid	1/4	3/4 (<1%)				+		+	+						
	FVB-*Neu*	Solid	0/10	0													
Complex	FVB and B6D2-*Tgfa*	Macrocyst	6/8	0		+	+	+++		+	+					+	
	FVB and B6D2-*Tgfa*	Lactation plaque	3/3	0		++	+	+++		++	+					+	
	BALB/cJ	Adenomyoepithelioma	5/5	0	+	+++	++	++		+++	++						
	BALB/cJ (mostly)	Adenoacanthoma	7/7	0	++	++	++	++	++	+++	+++	++	++	+	+	+	
	C3H/HeJ	Microacinar	8/8	0		++	+	++		++	+						
	BALB/cJ, C3H/HeJ	Papillary, 2 cells thick	2/2	0		++		++	+	++	++					+++	
	C3H/HeJ	Type P tumor	9/9	0		++	+	++		++	++		+			+++	
	FVB-*Wnt1*	Type P tumor	6/6	0		++	++	+++		++	++	+	+			++	
	B6SJL-*Wnt1*	Type P tumor	7/7	0		+	+	+		++	+					++	
EMT	C57BL6/J-Tag	Glandular with EMT	2/3	3/3 (6%)				+++		+++	++					++	+
	FVB-*Myc*	Glandular with EMT	4/6	6/6 (20%)		+	+	++		++	++					+	+
	PL/J and SJL/J	Glandular with EMT	4/4	4/4 (38%)		++	+	+++		++	++		+			++	++
	FVB-*Hras*	Solid with EMT	3/4	3/4 (<1%)		+		++		+	+						+

^a^data are coded, with "+++" representing highest grades; the intensity of immunolabeling was scored 0–3 (immunolabeling levels: 0 = none; 1 = weak; 2 = moderate; 3 = intense); the grade for each cell type was the product of average labeling intensity and the relative percentage of cells (0: 0; ≤ 1%: 1; 2–5%: 2; 6–25%: 3; 26–50%: 4; >50%: 5) labeled at the predominant intensity; outcome for each neoplasm was the sum of the grades for each cell type and the data presented are the average for each group; white boxes indicate an average grade <1; "+" indicates an average grade ≥ 1 and <5; "++" indicates an average grade ≥ 5 and <10; "+++" indicates an average grade ≥ 10; EMT, epithelial to mesenchymal transition; FLG, filaggrin; IVL, involucrin; K1, keratin 1; K5, keratin 5; K6, keratin 6; K8/18, keratins 8 and 18; K10, keratin 10; K17, keratin 17; LOR, loricrin; THH, trichohyalin; SMA, α-smooth muscle actin; VIM, vimentin.

^b^Absence of information for the transgene indicates that the tumor arose in a non-transgenic mouse.

^c^Based on the recommendations of the Annapolis meeting [17–19].

^d^Tumors with myoepithelial differentiation/tumors examined; carcinomas with minimal myoepithelial differentiation arising in mice transgenic for *Myc *were categorized as "simple carcinomas" because myoepithelial differentiation was interpreted as evidence of early EMT.

^e^Tumors with EMT/ tumors examined; the data in parenthesis indicate the average percentage of the tumors comprised of neoplastic cells with a mesenchymal phenotype.
